# RhMYB108, an R2R3-MYB transcription factor, is involved in ethylene- and JA-induced petal senescence in rose plants

**DOI:** 10.1038/s41438-019-0221-8

**Published:** 2019-12-01

**Authors:** Shuai Zhang, Qingcui Zhao, Daxing Zeng, Jiehua Xu, Hougao Zhou, Fenglan Wang, Nan Ma, Yonghong Li

**Affiliations:** 1 School of Applied Chemistry and Biological Technology, Postdoctoral Innovation Practice Base, Shenzhen Polytechnic, Shenzhen, Guangdong 518055 China; 2 Shenzhen Key Laboratory of Fermentation, Purification and Analysis, Shenzhen Polytechnic, Shenzhen, 518055 Guangdong China; 3 College of Horticulture and Landscape Architecture, Zhongkai University of Agriculture and Engineering, Guangzhou, Guangdong 510642 China; 4 China Beijing Key Laboratory of Development and Quality Control of Ornamental Crops, Department of Ornamental Horticulture, China Agricultural University, Beijing, China

**Keywords:** Plant signalling, Plant hormones

## Abstract

Rose (*Rosa hybrida*) plants are major ornamental species worldwide, and their commercial value greatly depends on their open flowers, as both the quality of fully open petals and long vase life are important. Petal senescence can be started and accelerated by various hormone signals, and ethylene is considered an accelerator of petal senescence in rose. To date, however, the underlying mechanism of signaling crosstalk between ethylene and other hormones such as JA in petal senescence remains largely unknown. Here, we isolated *RhMYB108*, an R2R3-MYB transcription factor, which is highly expressed in senescing petals as well as in petals treated with exogenous ethylene and JA. Applications of exogenous ethylene and JA markedly accelerated petal senescence, while the process was delayed in response to applications of 1-MCP, an ethylene action inhibitor. In addition, silencing of *RhMYB108* alter the expression of SAGs such as *RhNAC029*, *RhNAC053*, *RhNAC092*, *RhSAG12*, and *RhSAG113*, and finally block ethylene- and JA-induced petal senescence. Furthermore, RhMYB108 was identified to target the promoters of *RhNAC053*, *RhNAC092*, and *RhSAG113*. Our results reveal a model in which *RhMYB108* functions as a receptor of ethylene and JA signals to modulate the onset of petal senescence by targeting and enhancing senescence-associated gene expression.

## Introduction

Petals are a key component of flowers, and by attracting pollinators, they play a vital role in ensuring successful pollination^1^. Petal senescence is a natural and irreversible process that occurs at the terminal stage of flower development and is tightly controlled by environmental and developmental signals. Many physiological activities occur together with petal senescence, such as the breaking down of intracellular structures, the degrading of membrane systems and macromolecules, and the recycling of nutrients, all of which similarly occur together with leaf senescence^2,3^. Many excellent studies have reported that multiple vital activities are involved in petal senescence, such as programmed cell death (PCD)^4–6^, sugar metabolism^7^, hormone regulation^8,9^, and the regulatory activity of associated gene networks^10–12^.

The onset of petal senescence is initiated by external and internal cues, and phytohormones have been revealed to act as essential signals in initiating and modulating the senescence process. Among the hormones involved, ethylene and abscisic acid (ABA) function as inducers, and salicylic acid (SA), cytokinin (CTK), and gibberellic acid (GA) function as inhibitors^13–16^. The role of jasmonic acid (JA) and auxin in petal senescence is still unknown^10^.

Ethylene is considered a major endogenous signal of fruit ripening and leaf and petal senescence. Exogenous applications of ethylene can significantly accelerate petal senescence^13^ and floral organ abscission^17^. *ETHYLENE INSENSITIVE 2* (*EIN2*), which functions as the core member of ethylene signaling, acts as a key component in ethylene signaling. In *ein2-1* mutant plants, petal senescence and floral organ abscission are unaffected by exogenous ethylene applications. During petal senescence in rose, expression of the ethylene biosynthesis genes *1-AMINOCYCLOPROPANE-1-CARBOXYLIC ACID SYNTHASE 1* and *1-AMINOCYCLOPROPANE-1-CARBOXYLIC ACID SYNTHASE 2* (*ACS1* and *ACS2*, respectively) strongly increases, coupled with an accelerated increase in ethylene content^18^. It has been widely reported that JA functions in leaf senescence^19–22^, but its function in petal senescence is largely unclear. Some research has confirmed that applications of methyl jasmonate (MeJA), either as an aqueous solution or as a gas, accelerate petal senescence in *Petunia* and *Dendrobium*^23^; additional evidence has shown that, by completely suppressing tepal wilting, MeJA delays senescence in cut *Iris* flowers^24^.

Recent research on the ethylene and JA signaling pathways has shed light on the functional relationship of these two hormones in floral organ abscission^17^ and leaf senescence^25^. Floral organ abscission is a typical characteristic that occurs in response to ethylene. In *ein2-1* mutants, floral organ abscission was shown to be unaffected by applications of exogenous ethylene, whereas applications of JA could rescue the ethylene response of those mutants^17^. These results suggest that interplay between JA and ethylene occurs. In Arabidopsis leaf senescence, the function of JA in accelerated senescence depends on the ethylene signaling pathway, especially the key molecular components *ETHYLENE INSENSITIVE 3* (*EIN3*) and *ETHYLENE INSENSITIVE 3-LIKE 1* (*EIL1*). Studies have shown that the *ein3* mutant displays delay JA-induced leaf senescence^25^. *EIN3*/*EIL1* are the direct targets of JAZ repressors, which inhibit *EIN3* and *EIL1* transcriptional activities via physical interactions. Moreover, JASMONATE-ZIM DOMAIN (JAZ) proteins recruit HISTONE DEACETYLASE 6 (HDA6), a positive regulator of JA signaling, which subsequently inhibits *EIN3*/*EIL1*-dependent transcription and JA signaling because of acetylation modification^26^. However, understanding of the signaling crosstalk between ethylene and JA during petal senescence is unclear. In *Dendrobium* and *Petunia*, the effects of JA-induced tepal/petal senescence are accompanied by a massive production of ethylene, and the effects on senescence can be abolished by treatment with antagonists of ethylene synthesis or ethylene action^23^. This evidence reveals that the JA signal appears to be dragged in the ethylene pathway to modulate senescence.

Typical signs of petal senescence include color change, wilting, and petal abscission^8,10,18^. In this study, we identified a MYB transcription factor, RhMYB108, as being involved in the interplay between ethylene and JA signaling and thus regulating petal senescence in rose. According to their number of conserved MYB structural motifs, MYB proteins are classified into R1-MYB, R2R3-MYB, R3-MYB, and R4-MYB subfamilies^27,28^ and function in the regulation of plant growth, hormone signal transduction, stress and disease resistance, and secondary metabolism^29–33^. Here, we show that RhMYB108, which belongs to the R2R3-MYB family, is intensely expressed in rose petals. Silencing of *RhMYB108* delayed petal senescence by directly regulating the expression of senescence-associated genes (SAGs), including *RhNAC053, RhNAC092*, and *RhSAG113*, by physically interacting with their promoter; thus, RhMYB108 functions in petal senescence. In brief, our findings highlight the importance of RhMYB108 in the onset of petal senescence and imply that its function may integrate ethylene and JA signaling crosstalk during petal senescence.

## Materials and methods

### Plant materials and treatments

*R. hybrida cv.* Samantha plantlets were propagated as previously described^15,34^. Rooted rose and tobacco (*Nicotiana benthamiana*) plants were transplanted into pots and then grown in a growth chamber whose conditions included a 22 ± 1 °C temperature, a 16 h/8 h photoperiod and ~60% relative humidity.

With respect to JA treatment, flower buds of rose plantlets or petal discs were sprayed with 100 μM MeJA, after which they were untouched for 24 h; the control samples were placed in 0.1% ethanol. With respect to ethylene or 1-MCP treatment, rose plantlets or petal discs were exposed to 10 μL/L gaseous ethylene or 2 µL/L 1-MCP, respectively, in an airtight growth container for 24 h, and NaOH solution (1 M) was also added to the container to prevent CO_2_ accumulation, as previously described^15,18,35^.

### Measurement of ion leakage rates

Detached rose petals or petal discs were placed in 50 -mL tubes that contained 20 mL of deionized water. After the tubes were shaken at 28 °C for 30 min, the initial conductivity of the solution (C1) was measured using a conductivity meter (DDBJ-350, Precision & Scientific Instruments Co., Shanghai, China). The tubes were subsequently boiled in the same deionized water for 15 min, and the conductivity of the resulting solution (C2) was determined after the tubes cooled. The ion leakage rates were calculated by the C1:C2 ratios.

### RNA extraction and quantitative real-time PCR

RNA was extracted from the roots, stems, leaves, petals, sepals, and receptacles of rose plants as previously described^36^. The total RNA of the stamens and pistils was isolated using an RNA extraction kit (RN38-EASY, Aidlab Biotech, Beijing, China). The RNA was then reverse-transcribed using QRT SuperMix for qPCR (R233-01, Vazyme Biotech Co., Nanjing, China) and oligo (dT) primers. In conjunction with 2 μL of cDNA used as a template, qRT-PCR was performed via a Real-Time PCR System (Applied Biosystems, CA, USA) together with a Kapa SYBR Fast Universal qPCR Kit (Kapa Biosystems, Boston, MA, USA). *RhUBI2* was used as an internal control.

### Protein subcellular localization assays

With respect to subcellular localization assays, the open-reading frame (ORF) of RhMYB108 without the stop codon was amplified by PCR and then fused into the N-terminal region of a GFP label; the fusion protein was driven by the Super promoter. On the 3rd day after infiltration of *Agrobacterium* carrying the corresponding plasmid, the tobacco leaves were monitored by laser confocal fluorescence microscopy (Olympus Fluoview FV1000). pSuper::*GFP* served as a negative control, and pSuper::*NF-YA4-mCherry* was used as a nuclear marker.

### Transactivation and dual-luciferase reporter assays

Transcriptional activity assays of RhMYB108 were performed in tobacco leaves. The ORF of *RhMYB108* was amplified and inserted into a pBD vector, which served as an effector. A double-reporter vector, which contained a GAL4-LUC and an internal control REN gene driven by the 35S promoter, was also used.

To analyze the interaction between RhMYB108 and the SAG promoter in *N. benthamiana*, we constructed two vectors: pGreenII 0800-LUC and pGreenII 0029 62-SK^37–39^. The SAG promoters were amplified and inserted into the pGreenII 0800-LUC vector at the *Bam*HI and *Nco*I sites to drive the expression of the LUC reporter gene, and the *RhMYB108* ORF was inserted into the pGreenII 0029 62-SK vector at the *Bam*HI and *Kpn*I sites. The pGreenII 0800-LUC vector carries a *renilla luciferase* (REN) gene driven by the 35S promoter to serve as an internal control.

The effector construct and reporter plasmid were cotransformed into tobacco leaves by *Agrobacterium* infiltration as previously described^40^. At 48 h after transformation, the leaves were maintained in darkness for 5 min to measure LUC fluorescence.

### Yeast one-hybrid assays

Y1H assays were performed as described in the Yeast Protocols Handbook (Clontech Laboratories, USA). Two vectors were constructed for use in this system: pLacZi and pJG4-5. SAG promoters were inserted into the pLacZi vector, and the *RhMYB108* ORF sequence was inserted into the pJG4-5 vector. The plasmid construct was subsequently transformed into yeast strain EGY48, which was then grown on SD plates that lacked uracil and tryptophan, but contained X-β-gal for blue/white colony screening.

### Virus-induced gene silencing

VIGS of *RhMYB108* in rose petal discs and plantlets was performed as previously described^15,41^. A specific fragment of *RhMYB108* (437 bp in length) was amplified and inserted into a pTRV2 vector by *Xba*I and *Kpn*I. The vectors were transformed into *A. tumefaciens* strain GV3101, and the transformed *A. tumefaciens* cells were cultured in LB medium (supplemented with 50 μg/mL kanamycin and 50 μg/mL rifampicin) on a rocking platform (200 rpm) at 28 °C for 18 h. The cells were harvested and resuspended in infiltration buffer (10 mM MgCl_2_, 200 mM acetosyringone, 10 mM MES, pH 5.6) to a final OD_600_ of ~1.5. The culture mixtures contained pTRV1- and pTRV2-*RhMYB108* at a ratio of 1:1 (v/v) or pTRV1 and pTRV2 (the negative control). The culture mixtures were incubated in the dark at room temperature for 3–4 h before infiltration.

With respect to VIGS in rose petal discs, the petals were collected at stage 2 of flower opening, and two 1 cm diameter discs were isolated from symmetrical locations of the petals using a hole punch. The plantlets were subsequently placed in deionized water for 1 day to equilibrate after multiplication by tissue culture as previously described^15^. The discs and plantlets were submerged in infiltration buffer and then exposed to a vacuum (−25 kPa) twice (each for 60 s). After the release of the vacuum, the discs and plantlets were briefly washed with deionized water and then incubated in the dark at 8 °C for 3 days. The phenotypes of the discs were observed daily until necrosis, and the flowers of the rose plantlets were monitored at various time points from stages 1 to 6.

### Sequence analysis

With respect to multiple sequence alignment, the amino acid sequences were aligned using ClustalW with the default parameters (https://www.genome.jp/tools-bin/clustalw), and figures were created using the BioEdit software package (version 7.2.5)^42^. With respect to phylogenetic tree analysis, the alignment results were computed using MEGA software (version 5.05) based on the neighbor-joining algorithm and the following parameters: 1000 bootstrap replicates, Poisson correction, pairwise deletion, and uniform rates.

### Statistical analyses

Statistical analysis of the data was performed via GraphPad Prism 8.0 (GraphPad Software Inc., USA; http://www.graphpad.com/). Two groups of data were compared using two-sided Student’s *t* tests (*P < 0.05; **P < 0.01; ***P < 0.001; ****P < 0.0001). The means of multiple groups of data were compared via one-way ANOVA and Bonferroni’s post hoc test, with *P* < 0.05 considered significant.

## Results

### Ethylene and JA accelerate the senescence of rose petals

**Fig. 1:**
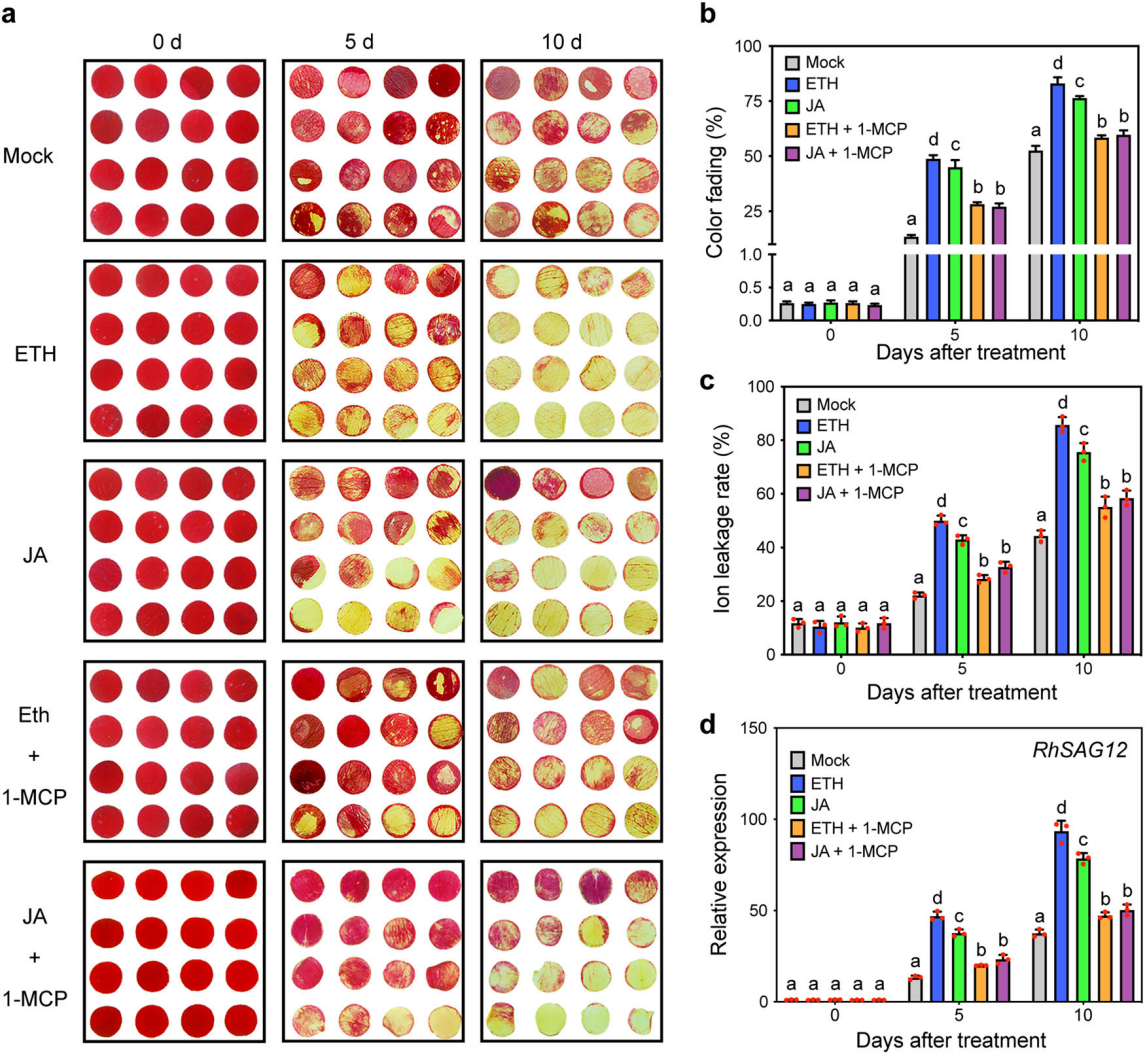
Effects of ethylene and JA on the senescence of rose petal discs. **a** Color fading phenotypes; **b** color fading rate; **c** ion leakage rate; and **d** *RhSAG12* expression levels in rose flower discs in response to ethylene, JA and 1-MCP treatment. ETH, 10 μL/L ethylene; JA, 100 μM MeJA; 1-MCP, 2 μL/L. The error bars represent the means of three biological replicates, and the letters indicate significant differences according to Duncan’s multiple range test (*P* < 0.05).

The function of ethylene and JA in the acceleration of leaf senescence has been widely reported^21,25^, but the interaction and crosstalk between these hormones during flower senescence has not been studied in detail. To determine the effects of ethylene and JA during flower senescence, we introduced a rose petal disc system^13,43^. As shown in Fig. 1a, the control petal discs at 10 days after incubation displayed slight color fading. After ethylene or JA treatment, the color fading started at 5 days, and nearly all discs showed severe color loss after 10 days of incubation. The color fading rate and ion permeability were markedly higher in the treated petals than in the control petals (Fig. 1b, c). Furthermore, the transcript levels of SAG *RhSAG12*, a molecular marker of rose petal senescence progression, were also significantly higher in the hormone-treated petal discs than in the control discs (Fig. 1d). In addition, compared with those treated with ethylene or JA, the petal discs treated with 1-MCP, a powerful antagonist of ethylene action, presented a slight color loss (Fig. 1a), and this result was supported by the experimental data concerning the color fading rate (Fig. 1b), ion leakage rate (Fig. 1c), and *RhSAG12* expression (Fig. 1d). Briefly, these results indicate that ethylene and JA accelerate the senescence process of rose petals and that the role of JA in petal senescence at least partly depends on ethylene signaling.

### Expression of RhMYB108 increased with petal senescence and was induced by ethylene and JA

Analysis of the information within the expressed sequence tag (EST) database of rose petals treated with exogenous ethylene (http://bioinfo.bti.cornell.edu/cgi-bin/rose_454/index.cgi.)^44^ revealed a transcript, RU04450, that encodes a putative MYB family transcription factor whose expression was markedly induced in response to the application of exogenous ethylene. The cDNA sequence of RU04450 is 1129 bp in length, with a 1032 bp ORF that encodes 343 amino acid residues. Phylogenetic analysis and sequence alignment revealed that RU04450 is closely related to *AtMYB108* from *Arabidopsis thaliana* (AT3G06490) (Supplementary Fig. 1a) and belongs to the R2R3-MYB family (Supplementary Fig. 1b); hence, the gene corresponding to RU04450 was named *RhMYB108*.

**Fig. 2:**
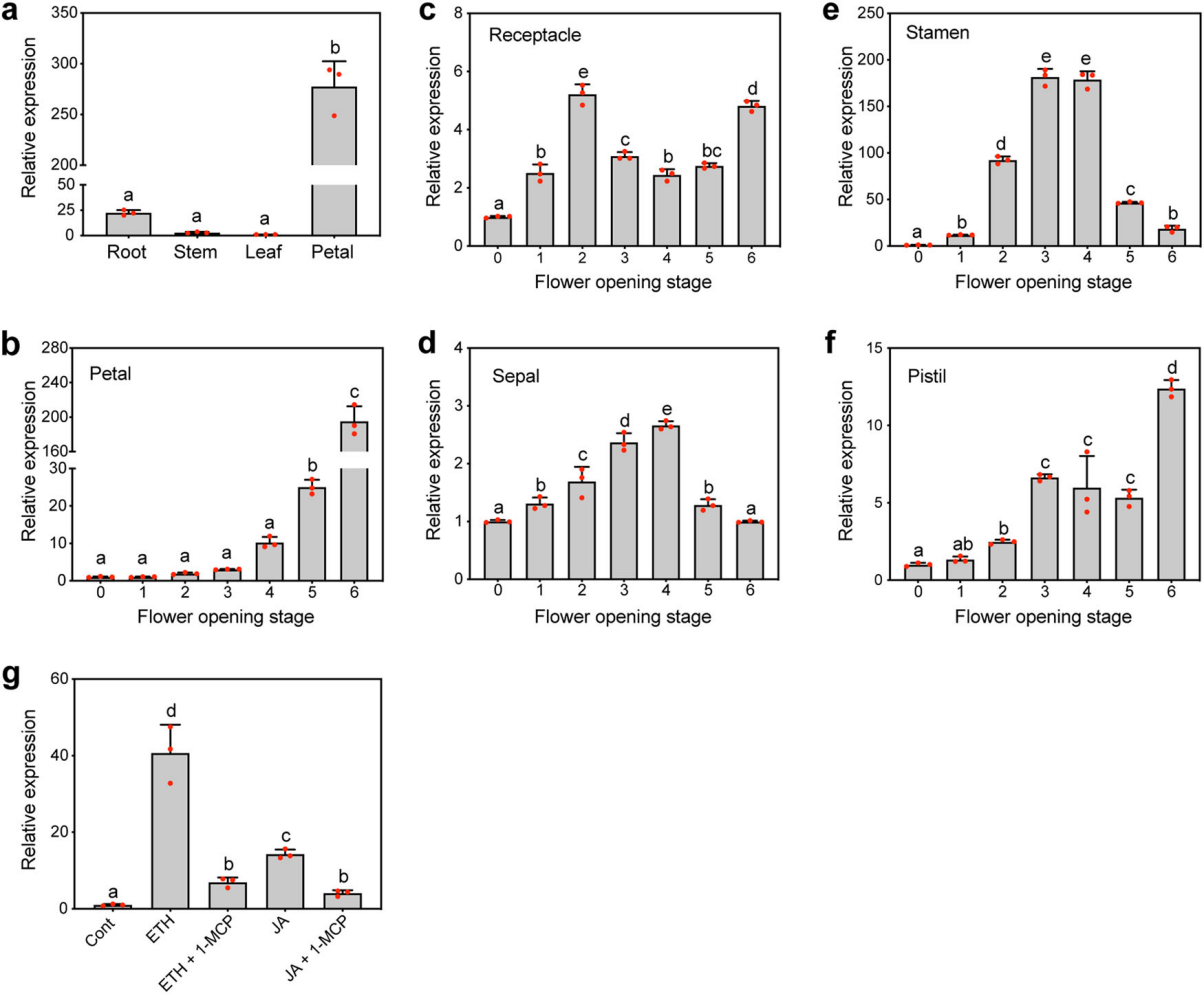
qRT-PCR analysis of the expression level of *RhMYB108*. **a** Expression profiles of *RhMYB108* in the roots, stems, leaves, and petals of rose plants. **b**, **c**, **d**, **e**, **f** Expression profiles of *RhMYB108* in the petals, receptacles, sepals, stamens, and pistils at various stages of flower opening. **g** Expression of *RhMYB108* in response to exogenous hormones. ETH, 10 μL/L ethylene; JA, 100 μM MeJA; 1-MCP, 2 μL/L. The petals at stage 2 were used as materials in **g**. *RhUBI2* was used as an internal control. Three independent biological repeats were tested, and the data are presented as the means ± SDs. The letters indicate significant differences according to Duncan’s multiple range test (*P* < 0.05).

To further investigate the function of *RhMYB108* during flower senescence, the transcripts of *RhMYB108* in all rose organs were measured by qRT-PCR. As shown in Fig. 2a, the expression of *RhMYB108* was greater in the rose petals than in the roots and stems, and was lowest in the leaves.

We further evaluated the expression of *RhMYB108* transcripts in different floral organs, including the petals, sepals, receptacles, stamens, and pistils, during rose flower opening. The expression of *RhMYB108* increased incrementally in the petals in parallel with flower opening (Fig. 2b), further implying that this gene may function in petal senescence. The *RhMYB108* expression pattern trends were similar between the sepals and stamens (Fig. 2d, e), and the transcript levels were higher in the partially opened flower buds (stages 3–4) than at other stages. In the receptacle and pistil, *RhMYB108* expression increased throughout the flower-opening process (Fig. 2c, f). In addition, we confirmed that petal senescence could be accelerated by exogenous applications of ethylene and JA. Expression of the *RhMYB108* transcript was consistently induced by treatment with exogenous ethylene and JA, and compared with that in response to applications of the control solution, its expression in response to applications of the ethylene analog 1-MCP was not altered (Fig. 2g). These data provide evidence of a correlation between *RhMYB108* expression and petal senescence.

### Silencing of RhMYB108 delays petal senescence

**Fig. 3:**
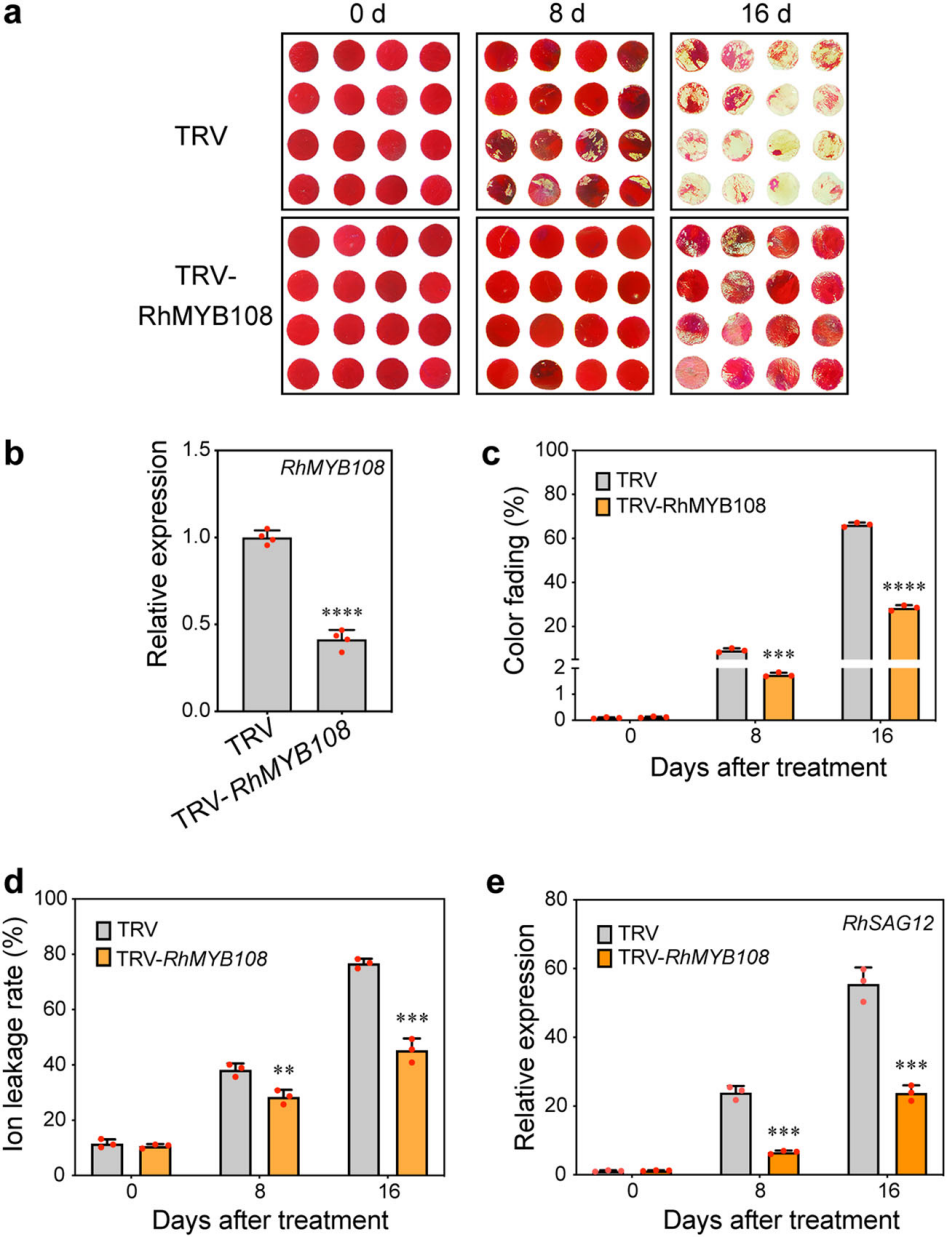
Silencing of *RhMYB108* delays senescence in rose petal discs. **a** Color fading phenotypes, **b** *RhMYB108* expression levels, **c** the color fading rate, **d** ion leakage rates, **e** *RhSAG12* expression levels were analyzed in *RhMYB108*-silenced and TRV control petal discs. The error bars represent the means of three biological replicates ± SDs, and the asterisks indicate significant differences according to Student’s *t* test (*P < 0.05; **P < 0.01; ***P < 0.001; ****P < 0.0001).

**Fig. 4:**
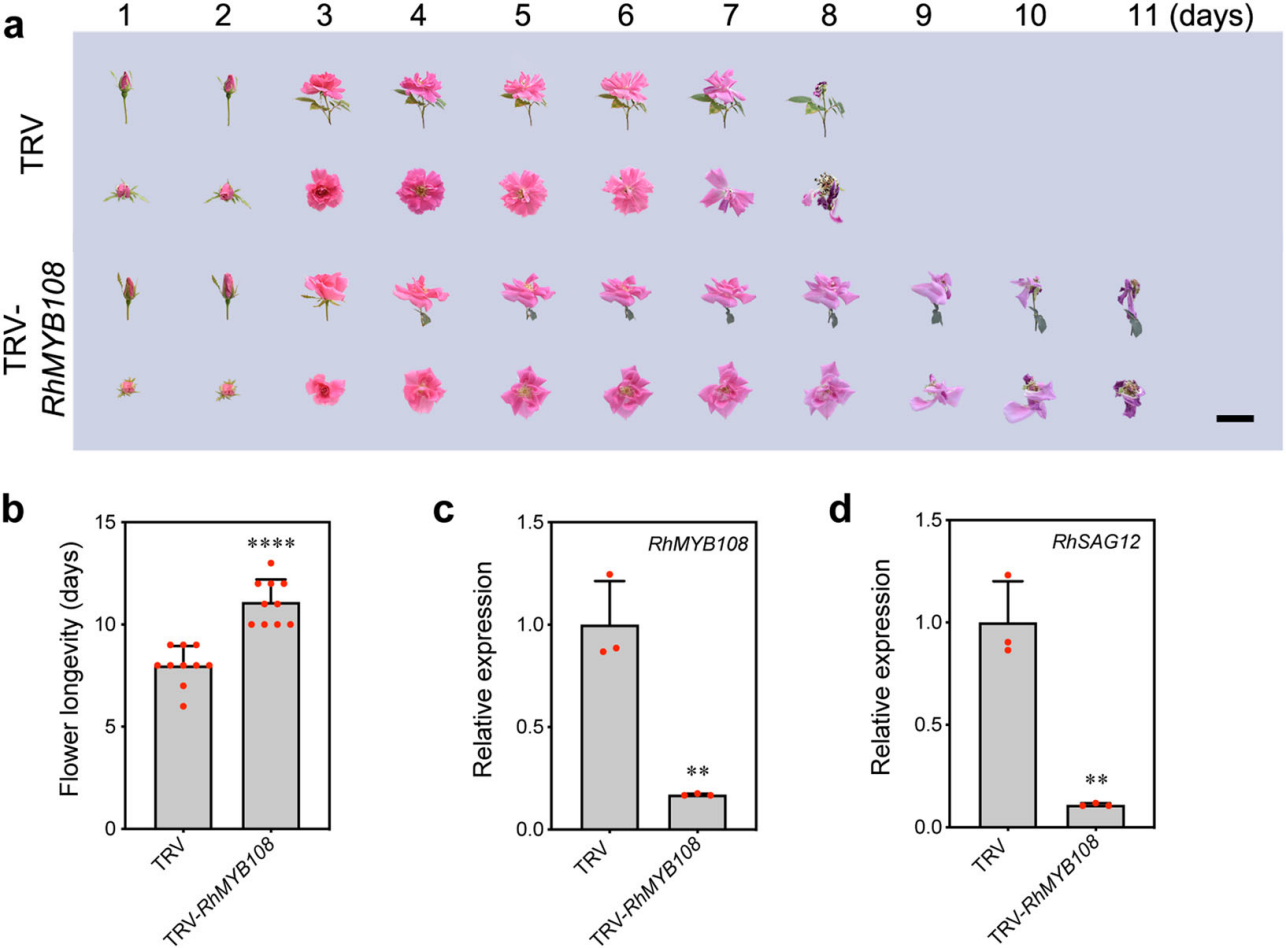
Silencing of *RhMYB108* delays petal senescence in rose plants. **a** The senescent phenotypes, **b** flower longevity, **c** *RhMYB108* expression levels, and **d** *RhSAG12* expression levels were analyzed in *RhMYB108*-silenced and TRV control plants. The results are the means of three biological replicates ± SDs, and the asterisks indicate significant differences according to Student’s *t* test (*P < 0.05; **P < 0.01; ***P < 0.001; ****P < 0.0001).

To assess the biological function of *RhMYB108* during the senescence of rose petals, we used VIGS specifically to silence *RhMYB108* transcripts in petal discs and rose plantlets; for this, we constructed a tobacco rattle virus vector (TRV-*RhMYB108*) from the 3′ end fragment of *RhMYB108* (431 bp in length). The rose petal disc assay revealed that color fading started at 8 days in the TRV control, and the discs were nearly discolored on day 16 (Fig. 3a). By contrast, the discs whose *RhMYB108* gene was silenced exhibited a markedly delayed senescent phenotype and displayed a slight color fading at 16 days. The expression of *RhMYB108* was significantly lower in the *RhMYB108*-silenced discs than in the TRV control discs (Fig. 3b). Consistent with this pattern, the silenced discs also presented significantly less color fading, ion leakage, and lower expression of *RhSAG12* than did the TRV control discs (Fig. 3c, d, e). With respect to the petal senescence process in rose plantlets, the *RhMYB108*-silenced plants also displayed a distinctly slower senescent phenotype than did the control plants (Fig. 4a); expression of *RhMYB108* and *RhSAG12* was markedly lower in the *RhMYB108*-silenced plants than in the TRV control plants (Fig. 4c, d). The flower life cycle of the *RhMYB108*-silenced plants was 11.1 ± 1.0 days, whereas that of the TRV control plants was 7.9 ± 0.9 days (Fig. 4b). These results indicate that *RhMYB108* functions as a positive regulator of the petal senescence process.

### Ethylene- and JA-triggered petal senescence is RhMYB108 dependent

We previously found that ethylene and JA could accelerate the senescence process of rose petals and that a reduction in *RhMYB108* expression led to a delayed-petal-senescence phenotype of *RhMYB108*-silenced plants. We therefore postulated that *RhMYB108* could also function in ethylene- and JA-induced petal senescence.

**Fig. 5:**
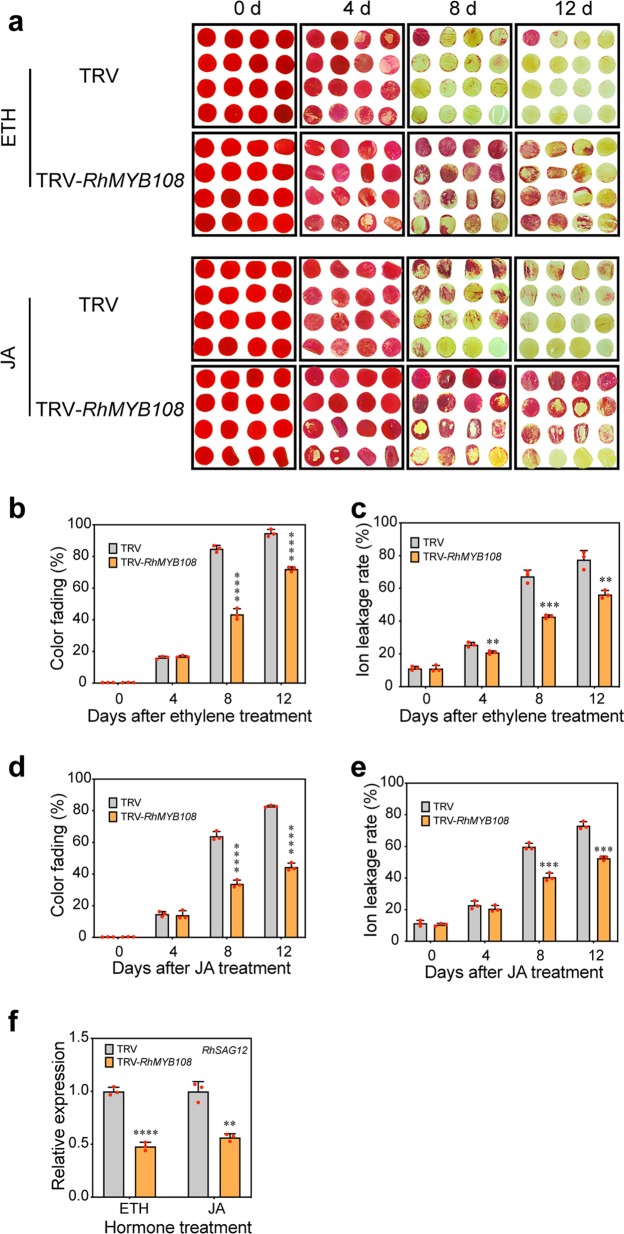
Effects of exogenous ethylene and JA treatment on the senescence of *RhMYB108*-silenced rose petal discs. **a** The phenotypes of rose petal discs treated with 10 μL/L ethylene or 100 μM MeJA were recorded. **b**, **c**, **d**, **e** Color fading and ion leakage rates (**b**, **c** ethylene treatment; **d**, **e** JA treatment) of *RhMYB108*-silenced and TRV control petal discs. **d** transcript levels of *RhSAG12* in *RhMYB108*-silenced and TRV control petal discs. Three biological replicates were tested, and the error bars represent the SDs. The asterisks indicate statistically significant differences (Student’s *t* test, *P < 0.05; **P < 0.01; ***P < 0.001; ****P < 0.0001).

**Fig. 6:**
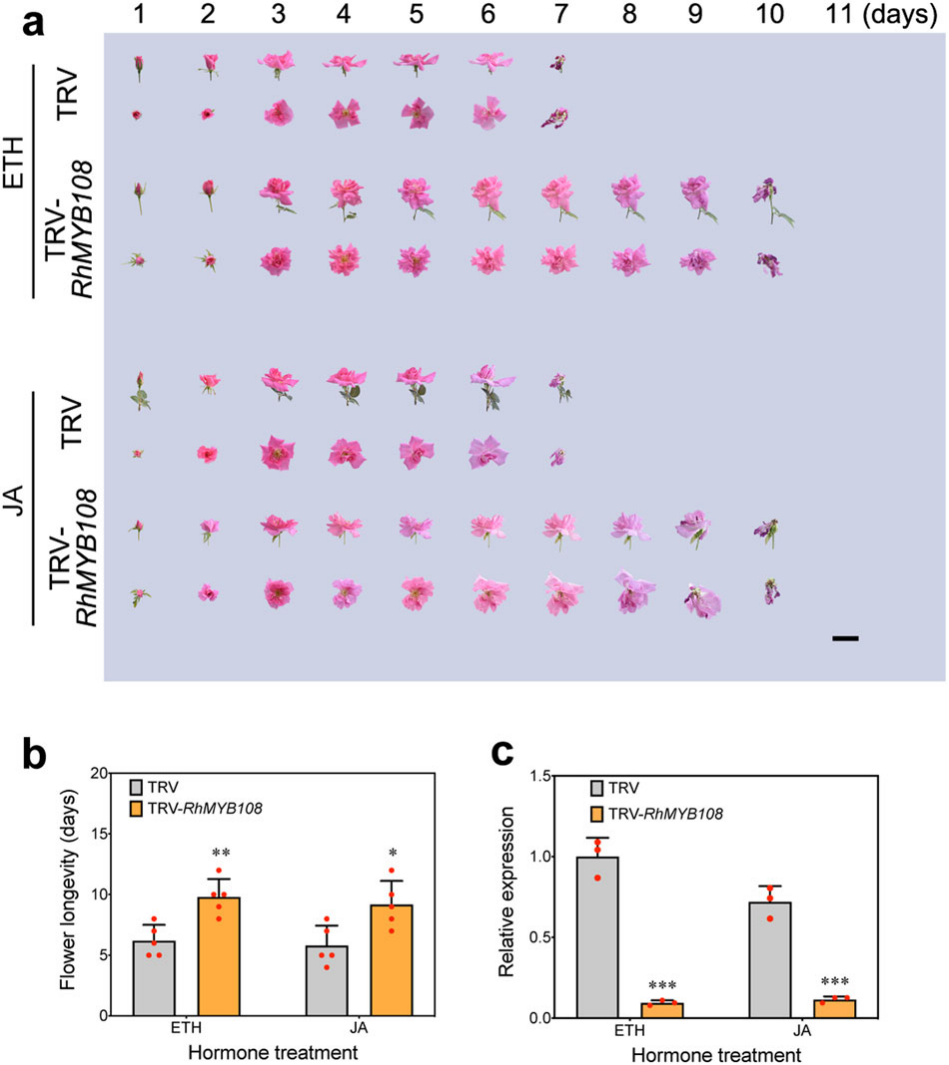
Effects of exogenous ethylene and JA treatment on the flower senescence of *RhMYB108*-silenced rose plants. **a** The flower senescent phenotypes of rose plants treated with 10 μL/L ethylene or 100 μM MeJA were recorded. **b**, **c** The flower longevity and transcript levels of *RhSAG12* in *RhMYB108*-silenced and TRV control rose plants treated with ethylene or JA. Three biological replicates were evaluated, and the error bars represent the SDs. The asterisks indicate statistically significant differences according to Student’s *t* test (*P < 0.05; **P < 0.01; ***P < 0.001; ****P < 0.0001).

To address this hypothesis further, we observed the phenotypes of *RhMYB108*-silenced petal discs or rose plantlets in response to applications of exogenous ethylene or JA. Compared with the TRV control discs, the *RhMYB108*-silenced discs presented a slightly senescent phenotype, which included mild color loss, markedly less ion leakage, and reduced *SAG12* expression, after the discs were in culture dishes for 8 days after ethylene or JA treatment. By day 12 after hormone treatment, a more extreme difference in the senescent phenotype emerged between the silenced discs and the TRV control discs (Fig. 5). With respect to the petal senescence process in rose plantlets, the phenotype of the *RhMYB108*-silenced plants revealed delayed ethylene- and JA-induced petal senescence (Fig. 6), which is consistent with the role of *RhMYB108* in the petal disc assay. These results indicate that *RhMYB108* accelerates the petal senescence process mediated by both ethylene and JA signaling.

### RhMYB108 directly targets senescence-related genes

To determine the subcellular localization of RhMYB108, we expressed an RhMYB108-GFP protein together with NF-YA4-mCherry (an mCherry-labeled nuclear marker protein) in *N. benthamiana* leaves, and both proteins were driven by the pSuper promoter. We observed a GFP fluorescence signal pattern that was consistent with the localization of the nuclear marker protein (mCherry) in the nucleus, indicating that RhMYB108 is a nuclear protein (Supplementary Fig. 2a).

To confirm whether RhMYB108 acts as a transcriptional regulator, we performed a transactivation assay in *N. benthamiana* leaves using the dual-luciferase reporter system. Compared with the pBD-negative control, the full-length (MYB108), middle-length (MYB108^1–284^), and short-length (MYB108^1–204^) RhMYB108 variants intensely activated the reporter gene (Supplementary Fig. 2b), and the relative LUC activity was 69.7-fold, 32.5-fold, and 10.7-fold higher than that of the negative control, respectively (Supplementary Fig. 2c). These results indicate that RhMYB108 functions as a transcriptional activator and that the C-terminus has high transactivation activity.

**Fig. 7:**
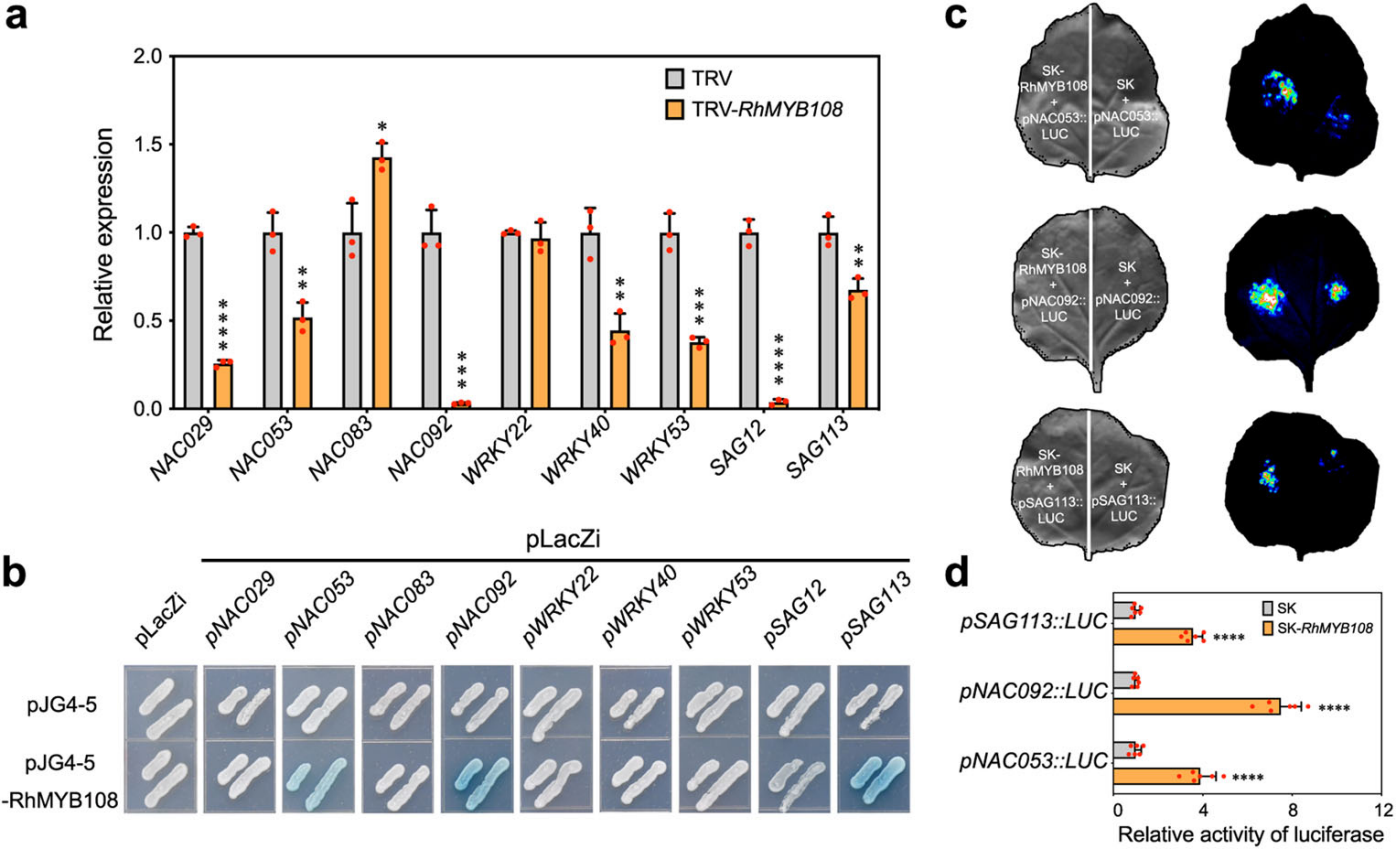
RhMYB108 directly binds to the promoter of *RhNAC053, RhNAC092* and *RhSAG113*. **a** qRT-PCR analysis of SAG expression in *RhMYB108-*silenced petals. **b** Interaction of RhMYB108 protein with the SAG promoter regions, as revealed using yeast one-hybrid assays. Interactions were determined based on yeast cell growth and were confirmed by the color indication of X-β-gal on SD/-Trp/-Ura medium plates. **c**, **d** Images of firefly luciferase fluorescence signals and relative reporter activity (LUC/REN) in *N. benthamiana* leaves. RhMYB108 protein was separately coexpressed with *pNAC053::LUC*, *pNAC092::LUC* and *pSAG113::LUC* in tobacco leaves. After 48 h of *Agrobacterium tumefaciens* infiltration, live LUC images and relative LUC activity (LUC/REN) in tobacco leaves were assayed. The error bars represent the means of six biological replicates, and the asterisks indicate statistically significant differences according to Student’s *t* test (*P < 0.05; **P < 0.01; ***P < 0.001; ****P < 0.0001).

SAGs usually serve as markers of the senescence process. Here, a series of SAG expression levels were detected using qRT-PCR in *RhMYB108*-silenced plants and TRV control plants. As expected, the expression of only *NAC083* was obviously upregulated, acting as a senescence-inhibiting regulator^45^ when *RhMYB108* was silenced. The expression trend of *WRKY22* was markedly similar in both the *RhMYB108*-silenced plants and the TRV control plants, while the expression of the transcripts of *NAC029*, *NAC053*, *NAC092*, *WRKY40*, *WRKY53*, *SAG12*, and *SAG113* was notably inhibited in the *RhMYB108*-silenced plants (Fig. 7a). These data confirmed that by regulating the expression of SAGs, *RhMYB108* may act as a transcriptional activator to accelerate petal senescence.

To better understand the possible regulatory mechanism that governs how *RhMYB108* regulates petal senescence in rose plants, the promoter region of SAGs, including the 1.5- kb region upstream of the initiation codon, was amplified and inserted into a pLacZi vector, and the ORF of *RhMYB108* was amplified and inserted into a pJG4-5 plasmid, yielding an expression vector. Y1H assays revealed that the RhMYB108 protein could bind to the promoter region of *NAC053*, *NAC092*, and *SAG113*, triggering the expression of the LacZ reporter gene (Fig. 7b). Additional evidence from a dual-luciferase reporter assay in *N. benthamiana* revealed that the activity of the promoters of *NAC053*, *NAC092*, and *SAG113* could be induced by overexpression of *RhMYB108* driven by the CaMV 35S promoter (Fig. 7c, d). Together, these results clarify that by directly altering SAG expression, RhMYB108 functions in the acceleration of petal senescence.

## Discussion

### RhMYB108 positively regulates petal senescence in rose

Petal senescence is a highly programmed event that follows visible signs, including color fading, wilting, and abscission^13^. At the transcriptional level, the expression of many transcription factors is altered during senescence, implying that complex regulatory networks are involved in this process^46,47^. As members of one of the most important families of transcription factors in plants, MYB proteins play pivotal roles in modulating multiple developmental and physiological processes. In Arabidopsis, only a few MYB transcription factors, such as MYBL^48^, MYBH^49^, and MYB2^50^, are reportedly involved in the modulation of the senescence process. We found that *RhMYB108* transcripts were more highly expressed in the petals than in other organs, including the roots, stems, and leaves (Fig. 2a). In addition, *RhMYB108* expression was higher in senescent petals than in young petals during flower opening (Fig. 2b). These data implied that *RhMYB108* may play a role in petal senescence. Furthermore, compared with the TRV control plants, the *RhMYB108-*silenced plants exhibited delayed petal senescence (Figs. 3, 4), along with less ion leakage and lower *SAG12* expression. The expression of several SAGs, such as *SAG12*, *SAG113*, *NAC029*, *NAC053*, and *NAC092*, which serve as molecular markers of the senescence process, was downregulated in the *RhMYB108*-silenced lines (Fig. 7a). Additional research showed that these SAGs contain some MYB-specific cis-elements in their promoters, and our Y1H and dual-luciferase reporter assays demonstrated that these SAGs (*NAC053*, *NAC092*, and *SAG113*) were direct targets of RhMYB108 (Fig. 7b, c, d).

Several previous studies have demonstrated that, by acting downstream of AtMYB21, *AtMYB108* can function at the transcriptional level in Arabidopsis stamen and pollen maturation in response to JA^51^. *AtMYB108* (*AtBOS1*) also acts as a negative regulator in controlling wound-induced cell death mediated by ABA signaling^52^. Recently, by targeting the *AtNAC003* promoter during dark-stressed leaf senescence, *AtMYB108* was shown to directly control *AtNAC003* expression^53^. These reports reveal that the MYB108 transcription factor participates in complex signaling and regulatory networks mediated by multiple phytohormone pathways and stress responses.

### RhMYB108 mediates ethylene and JA signaling during senescence

Multiple phytohormones play a role in regulating the process of petal senescence. Among these hormones, ethylene is generally considered a senescence accelerator on the basis of increased ethylene production during petal senescence. The function of JA in leaf senescence has been widely investigated^19,21,22,54^; however, its role in petal senescence is currently unclear, and the detailed mechanism by which signaling crosstalk between ethylene and JA modulates petal senescence is still poorly understood.

Studies have shown that endogenous JA levels are stable during flower opening or senescence in orchids and in *Lilium*^8,55,56^. Another study showed that applications of endogenous JA could delay tepal senescence in *Iris* flowers by 2 days^57^. In contrast, treatment of *Dendrobium* and *Petunia* flowers with JA reportedly accelerated petal senescence by promoting ethylene production. In addition, applications of silver thiosulfate, an inhibitor of ethylene action, completely inhibited the effects of JA function on accelerating petal senescence^23^. In conclusion, the role of JA in petal senescence seems contradictory, and additional investigations are needed to reveal the definite mechanisms underlying JA-mediated petal senescence. Here, our results are consistent with those of the study by Porat et al.^23^ and further verify the positive effects of JA on petal senescence. In addition, the function of JA in accelerating the senescence process was abolished by applying 1-MCP, an ethylene action inhibitor (Fig. 1). Our data imply that JA may act as a promoter of petal senescence via the ethylene signaling pathway. The signaling crosstalk between ethylene and JA may depend on the interaction of JAZ-EIN3 and JAZ-EIL1. A previous paper revealed that JA could enhance the transcriptional activity of EIN3/EIL1 by removing JAZ proteins, which physically interact with and inhibit *EIN3*/*EIL1* transcription and JA signaling by recruiting the corepressor HDA6, an RPD3-type histone deacetylase that functions in modulating histone acetylation^26^.

qRT-PCR analysis revealed that the expression of *RhMYB108* transcripts increased in response to exogenous ethylene and JA treatment (Fig. 2g), and the results implied that *RhMYB108* could be involved in the ethylene and JA signaling pathways at the transcriptional level. Overall, RhMYB108 may function as a member of the regulatory network that integrates ethylene and JA signaling to modulate the onset and progression of petal senescence. In the future, it will be interesting to investigate how ethylene and JA signaling regulates RhMYB108 transcription, which would provide further insights into the detailed mechanism that governs signaling crosstalk between ethylene and JA during petal senescence.

In this study, we identified a MYB transcription factor, RhMYB108, as being highly expressed in senescing petals as well as in petals treated with exogenous ethylene and JA. RhMYB108 belongs to the R2R3-MYB family and is closely related to AtMYB108 from *Arabidopsis thaliana* (AT3G06490). Silencing of *RhMYB108* results in delayed petal senescence and blocks ethylene- and JA-induced petal senescence by altering the expression of SAGs, including *RhNAC029*, *RhNAC053*, *RhNAC092*, *RhSAG12*, and *RhSAG113*. Furthermore, RhMYB108 was identified as targeting the promoters of *RhNAC053*, *RhNAC092*, and *SAG113*. In brief, our findings highlight the importance of RhMYB108 in the onset of petal senescence and imply that its function may integrate ethylene- and JA signaling crosstalk during petal senescence.

## Supplementary information

Supplementary material

## Data Availability

The GenBank accession number of the RhMYB108 rose gene used in this study is MK606453.
