# Author Correction: RhMYB108, an R2R3-MYB transcription factor, is involved in ethylene- and JA-induced petal senescence in rose plants

**DOI:** 10.1038/s41438-019-0230-7

**Published:** 2019-12-27

**Authors:** Shuai Zhang, Qingcui Zhao, Daxing Zeng, Jiehua Xu, Hougao Zhou, Fenglan Wang, Nan Ma, Yonghong Li

**Affiliations:** 1 School of Applied Chemistry and Biological Technology, Postdoctoral Innovation Practice Base, Shenzhen Polytechnic, Shenzhen, Guangdong 518055 China; 2 Shenzhen Key Laboratory of Fermentation, Purification and Analysis, Shenzhen Polytechnic, Shenzhen, 518055 Guangdong China; 3 College of Horticulture and Landscape Architecture, Zhongkai University of Agriculture and Engineering, Guangzhou, Guangdong 510642 China; 4 China Beijing Key Laboratory of Development and Quality Control of Ornamental Crops, Department of Ornamental Horticulture, China Agricultural University, Beijing, China

**Keywords:** Plant signalling, Plant hormones

**Correction to:** Horticulture Research

10.1038/s41438-019-0221-8 Published online 01 December 2019

After publication of our article [1], we became aware that there were errors in Fig. 7b, namely the negative control of pLacZi + pJG4-5-RhMYB108 (2nd row, panel 1). The error does not affect the result, discussion or conclusion in the article. The correct version of Figure is shown below. We apologise to the journal and to readers for this error.

The original article has been corrected.

**Fig. 7:**
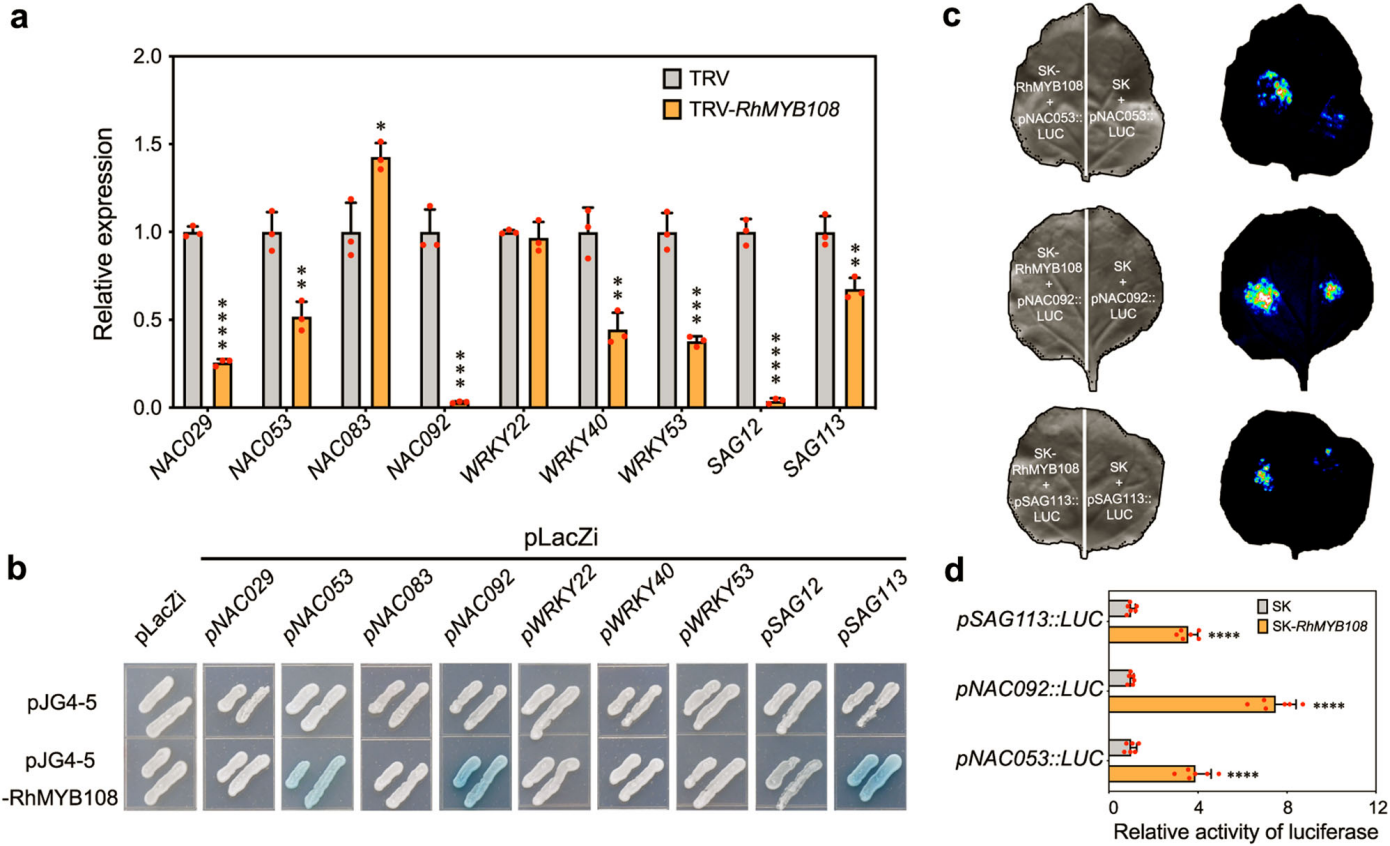
**a** qRT-PCR analysis of SAG expression in RhMYB108-silenced petals. **b** Interaction of RhMYB108 protein with the SAG promoter regions, as revealed using yeast one-hybrid assays. Interactions were determined based on yeast cell growth and were confirmed by the color indication of X-β-gal on SD/-Trp/-Ura medium plates. **c, d** Images of firefly luciferase fluorescence signals and relative reporter activity (LUC/REN) in *N. benthamiana* leaves. RhMYB108 protein was separately coexpressed with *pNAC053::LUC*, *pNAC092::LUC* and *pSAG113::LUC* in tobacco leaves. After 48 h of Agrobacterium tumefaciens infiltration, live LUC images and relative LUC activity (LUC/REN) in tobacco leaves were assayed. The error bars represent the means of six biological replicates, and the asterisks indicate statistically significant differences according to Student’s t test (*P < 0.05; **P < 0.01; ***P < 0.001; ****P < 0.0001).
