# Supplementary material for: RhMYB108, an R2R3-MYB transcription factor, is involved in ethylene- and JA-induced petal senescence in rose plants
Source: Hortic Res. 2019 Dec 1;6:131. doi: 10.1038/s41438-019-0221-8 (PMC6885062; doi:10.1038/s41438-019-0221-8)
Supplement: Supplementary file 1 — Supplementary material [file 41438_2019_221_MOESM1_ESM.docx]

**Supplementary Information**

**RhMYB108, an R2R3-MYB transcription factor, is involved in ethylene- and JA-induced petal senescence via in rose plants.**

**This PDF file includes:**

Supplementary Fig. 1 to 2

Supplementary Tables 1

Supplementary Figures and legends


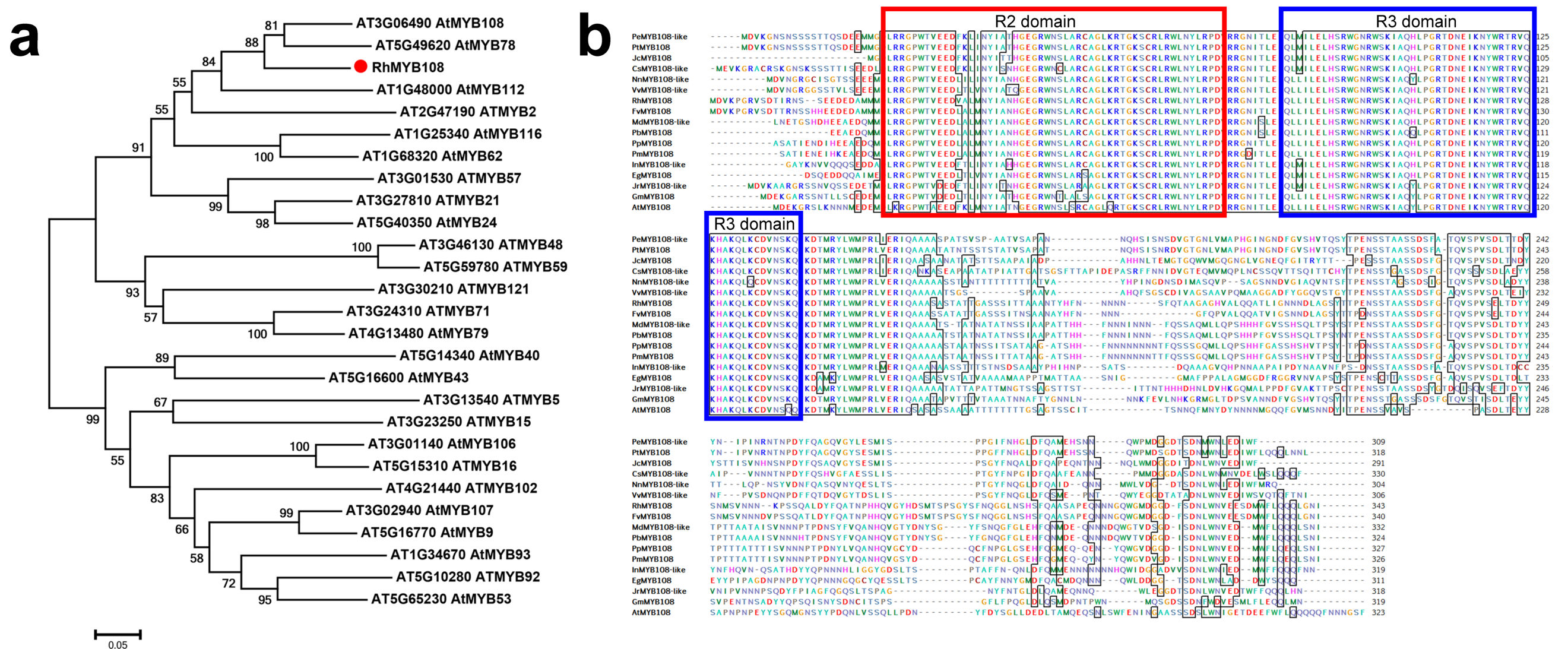


**Supplementary figure 1.** **Phylogenetic analysis and protein sequence alignment of *RhMYB108*. a** Phylogenetic analysis of *RhMYB108* and R2R3-type MYB genes from Arabidopsis. Bootstrap values indicate the confidence of each branch and the scale indicates branch length. **b** Sequence alignment of RhMYB108 protein and R2R3-type MYB proteins from other species. The conserved R2-MYB domain was marked by red square and R3-MYB domain by blue square. GenBank accession numbers are as follows: *Rosa hybrida* RhMYB108 (MK606453), *Fragaria vesca* FvMYB108 (XP_004295042), *Populus euphratica* PeMYB108-like (XP_011027944), *Malus domestica* MdMYB108-like (XP_008390410), *Populus trichocarpa* PtMYB108 (XP_002311369), *Pyrus* × *bretschneideri* PbMYB108 (XP_009372081), *Prunus persica* PpMYB108 (XP_007227100), *Jatropha curcas* JcMYB108 (XP_012084138), *Citrus sinensis* CsMYB108-like (XP_006488792), *Ipomoea nil* InMYB108-like (XP_019180052), *Eucalyptus grandis* EgMYB108 (XP_010024826), *Juglans regia* JrMYB108-like (XP_018825593), *Prunus mume* PmMYB108 (XP_008223427), *Nelumbo nucifera* NnMYB108-like (XP_010276490), *Vitis vinifera* VvMYB108-like (NP_001267991), *Glycine max* GmMYB108 (NP_001239915), *Arabidopsis thaliana* AtMYB108 (NP_187301).


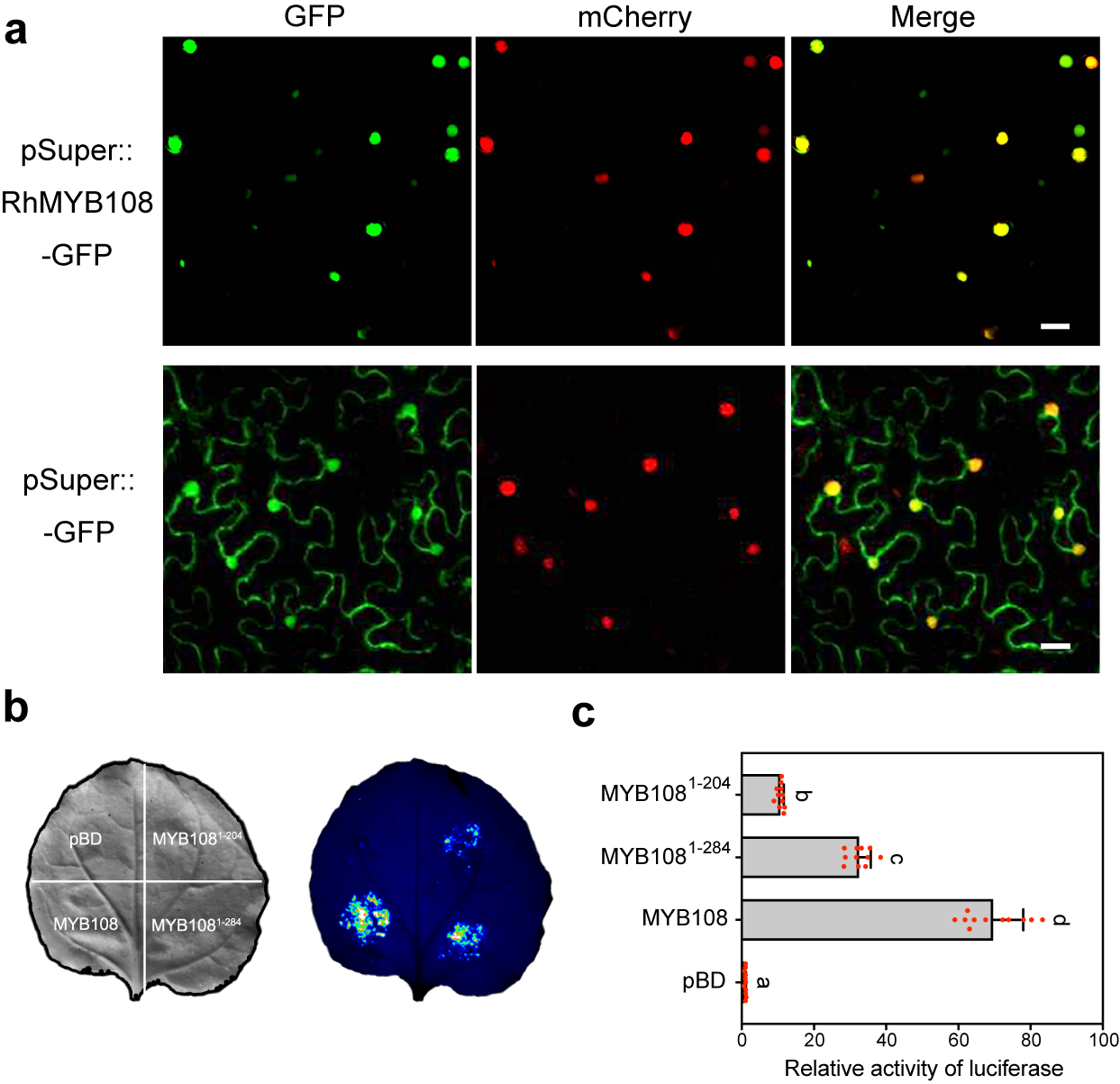


**Supplementary figure 2.** **Subcellular localization and transcriptional activity of RhMYB108. a** Subcellular localization of RhMYB108 in tobacco leaves. GFP fused to the C-terminal region of RhMYB108, and the fusion protein was driven by the Super promoter. An mCherry labeled fusion protein (NF-YA4-mCherry) was used as a nuclear marker, and pSuper::*GFP* was used as negative control. GFP and mCherry fluorescence signal were visualized by confocal microscopy 3 d after infiltration. Scale bar, 50 μM. **b**, **c** Transcriptional activation ability of RhMYB108 in tobacco leaves. The reporter plasmids contain 5× GAL4 and firefly luciferase (LUC) driven by mini CaMV35S, and *renilla* luciferase (REN) driven by CaMV35S. The effector plasmids contain the RhMYB108, RhMYB108^1-284^ or RhMYB108^1-204^, which fused to C-terminal of GAL4BD driven by the CaMV35S, respectively. After 48 h of the *Agrobacterium tumefaciens* infiltration, live LUC image (B) and relative LUC activity (ratio of LUC to REN) (C) in tobacco leaves were assayed. Error bar represents the means of five biological replicates, and the letters indicate significant differences according to Duncan's multiple range test (*P* < 0.05).

| **Supplementary Table 1. List of primers used.** | |
| --- | --- |
| **Primers** | **Sequence** |
| **For qRT-PCR analysis** | |
| *RhUBI2-*F | 5’-GCCCTGGTGCGTTCCCAACTG-3’ |
| *RhUBI2-*R | 5’-CCTGCGTGTCTGTCCGCATTG-3’ |
| *RhMYB108-*F | 5’-GTTGTAAAGCGACGTGTCCTG-3’ |
| *RhMYB108-R* | 5’-GCCGAGATTGGTCGAGAGAA-3’ |
| *RhWRKY53-*F | 5’-AAACATTGCAGTGAACGGGC -3’ |
| *RhWRKY53-*R | 5’-AGAAAAGTTCGGGAGCGTCA-3’ |
| *RhSAG12-F* | 5’-AGCGGAGAAGCCTTTCAGTC-3’ |
| *RhSAG12-R* | 5’-CAGCATGGTTCAGGCTGGTA-3’ |
| *RhNAC029-F* | 5’-GTTGCCGACCACAACAATGG-3’ |
| *RhNAC029-R* | 5’-GGGATGATGGAAACAGGGCA-3’ |
| *RhNAC083-F* | 5’-CAGAGCCCTGGACGTTTTGT-3’ |
| *RhNAC083-R* | 5’-TGGGTGTTCGAGAGAGACGA-3’ |
| *RhNAC053-F* | 5’-CCCACCAACAAAATGAGTCGT-3’ |
| *RhNAC053-R* | 5’-ACAGAATTAGGGTTTCCAATCCAGA-3’ |
| *RhNAC092-F* | 5’-AGATCCTCCTCGTCGAGTCA-3’ |
| *RhNAC092-R* | 5’-ACACACGAGGCTACATTCCA-3’ |
| *RhWRKY22-F* | 5’-TACCAAACCCAAGGGCTACTC-3’ |
| *RhWRKY22-R* | 5’-TCATCCGCCGTAGGACTCTG-3’ |
| *RhWRKY53-F* | 5’-AACCGATCAGGCCCTTGCT-3’ |
| *RhWRKY53-R* | 5’-ACGAGATTTCGAAGATTGACCCA-3’ |
| *RhSAG113-F* | 5’-TGTGTCCTCTGTAACCAACAAGT-3’ |
| *RhSAG113-R* | 5’-CTCCAACTAGACCGCAGCAA-3’ |
| **promoter clone** | |
| *pSAG12-F* | 5’-AGCAATTCCGAACAGAGCCT-3’ |
| *pSAG12-R* | 5’-AGTCGTAAGGGGACTCCTGG-3’ |
| *pSAG113-F* | 5’-GGATCCACGTCAGCTACCTC-3’ |
| *pSAG113-R* | 5’-GGCACTACTCTCGGTTGGAC-3’ |
| *pNAC092-F* | 5’-AGTTCGTGCCATACTTGGGT-3’ |
| *pNAC092-R* | 5’-GGCAACCTGGTGGATTGTTTC-3’ |
| *pNAC055-F* | 5’-TGCACCGTGACACGAAAGAT-3’ |
| *pNAC055-R* | 5’-TTCGTTAACGTGCAGCCTCA-3’ |
| *pNAC029-F* | 5’-TACAACCCCAATCCGAAGGC-3’ |
| *pNAC029-R* | 5’-TGTCAGCTAACCTCAACGGG-3’ |
| *pNAC053-F* | 5’-ACGTACAATTGGATCATGTGGA-3’ |
| *pNAC053-R* | 5’-CAGAATTAGGGTTTCCAATCCAG-3’ |
| *pNAC042-F* | 5’-GTGTCCAAAACAGCCAGACC-3’ |
| *pNAC042-R* | 5’-GAAGAGGGATTTGCTTGGTCA-3’ |
| *pNAC083-F* | 5’-TCGATGTCACGATTGTTCTAGT-3’ |
| *pNAC083-R* | 5’-GAGACGACCCATTTGGCGTA-3’ |
| ***RhMYB108* ORF clone** | |
| *RU04450-F* | 5’-CTCTTCAATCAGAGGCTTTT-3’ |
| *RU04450-R* | 5’-GGTCAAATATTGCCGAGTT-3’ |
| Super1300-F | 5'-GCCATTTCGCCTTTTCAG-3' |
| Super1300-R | 5'-TGATAATCATCGCAAGACCG-3' |
| **TRV vector construction** | |
| TRV1-F | 5'-TTACAGGTTATTTGGGCTAG-3' |
| TRV1-R | 5'-CCGGGTTCAATTCCTTATC-3' |
| TRV2-F | 5'-TGGGAGATGATACGCTGTT-3' |
| TRV2-R | 5'-CCTAAAACTTCAGACACG-3' |
| *RhMYB108-silencing-*F | 5’-TAGTCTAGAGCAGGACACGTCGCTTTA-3’ |
| *RhMYB108-silencing-*R | 5’-ATTCTCGAGCCGAGTTGCTGCTGTAAGA-3’ |
| **For yeast one-hybrid assay** | |
| *ol-PJG-MYB108-F* | 5’-attatgcctctcccgaattcATGGATGTCAAACCAGGAAG-3’ |
| *ol-PJG-MYB108-R* | 5’-gaagtccaaagcttctcgagTCAAATATTGCCGAGTTGCT-3’ |
| *ol-lacz-pNAC029-F* | 5’-cctttgatattggatcGAATTCtttcagtatgttgtatttag-3’ |
| *ol-lacz-pNAC029-R* | 5’-tacagagcacatgcctcgaggagttagtaattcagtttgg-3’ |
| *ol-lacz-pNAC083-F* | 5’-cctttgatattggatcGAATTCtgaaaggatgcttgtaatta-3’ |
| *ol-lacz-pNAC083-R* | 5’-tacagagcacatgcctcgagtccagggctctgcgaaaagg-3’ |
| *ol-lacz-pNAC042-F* | 5’-cctttgatattggatcGAATTCgggtgattcatcgtcacgtt-3’ |
| *ol-lacz-pNAC042-R* | 5’-tacagagcacatgcctcgagaaaccaaagaaaattattgt-3’ |
| *ol-lacz-pNAC053-F* | 5’-cctttgatattggatcGAATTCATCCTCTTAGGGTCTGTTTC-3’ |
| *ol-lacz-pNAC053-R* | 5’-tacagagcacatgcctcgagTTTTCAAATTGAGGTGGAGA-3’ |
| *ol-lacz-pNAC092-F* | 5’-cctttgatattggatcGAATTCGCTTACATCTCTATATCTCTAA-3’ |
| *ol-lacz-pNAC092-R* | 5’- tacagagcacatgcctcgagTTTTAACTTTTCTGTAATAAAT-3’ |
| **For dual luciferase reporter assay** | |
| *OL-LUC-pNAC053-F* | 5’-tcgacggtatcgataagcttATCCTCTTAGGGTCTGTTTC-3’ |
| *OL-LUC-pNAC053-R* | 5’-gctctagaactagtggatccTTTTCAAATTGAGGTGGAGA-3’ |
| *OL-LUC-pSAG113-F* | 5’-tcgacggtatcgataagcttCACGAAGCTGTTAACCTGCG-3’ |
| *OL-LUC-pSAG113-R* | 5’-gctctagaactagtggatccTCCTCGAAAGATTGAGAAGA-3’ |
| *ol-Luc-pNAC092-F* | 5’-tcgacggtatcgataagcttGCTTACATCTCTATATCTCTAA-3’ |
| *ol-Luc-pNAC092-R* | 5’-gctctagaactagtggatccTTTTAACTTTTCTGTAATAAAT-3’ |
| *EcoRI-pG62-108-F* | 5’-cgggctgcaggaattcatggatgtcaaaccag-3’ |
| *XhoI-pG62-108-R* | 5’-cgggccccccctcgagaatattgccgagttgc-3’ |
|  |  |
